# Supplementary material for: Functional response to a microbial synbiotic in the gastrointestinal system of children: a randomized clinical trial
Source: Pediatr Res. 2022 Nov 2;93(7):2005–13. doi: 10.1038/s41390-022-02289-0 (PMC10313516; doi:10.1038/s41390-022-02289-0)
Supplement: Supplementary file 3 — Supplementary Figure S2 [file 41390_2022_2289_MOESM3_ESM.pdf]

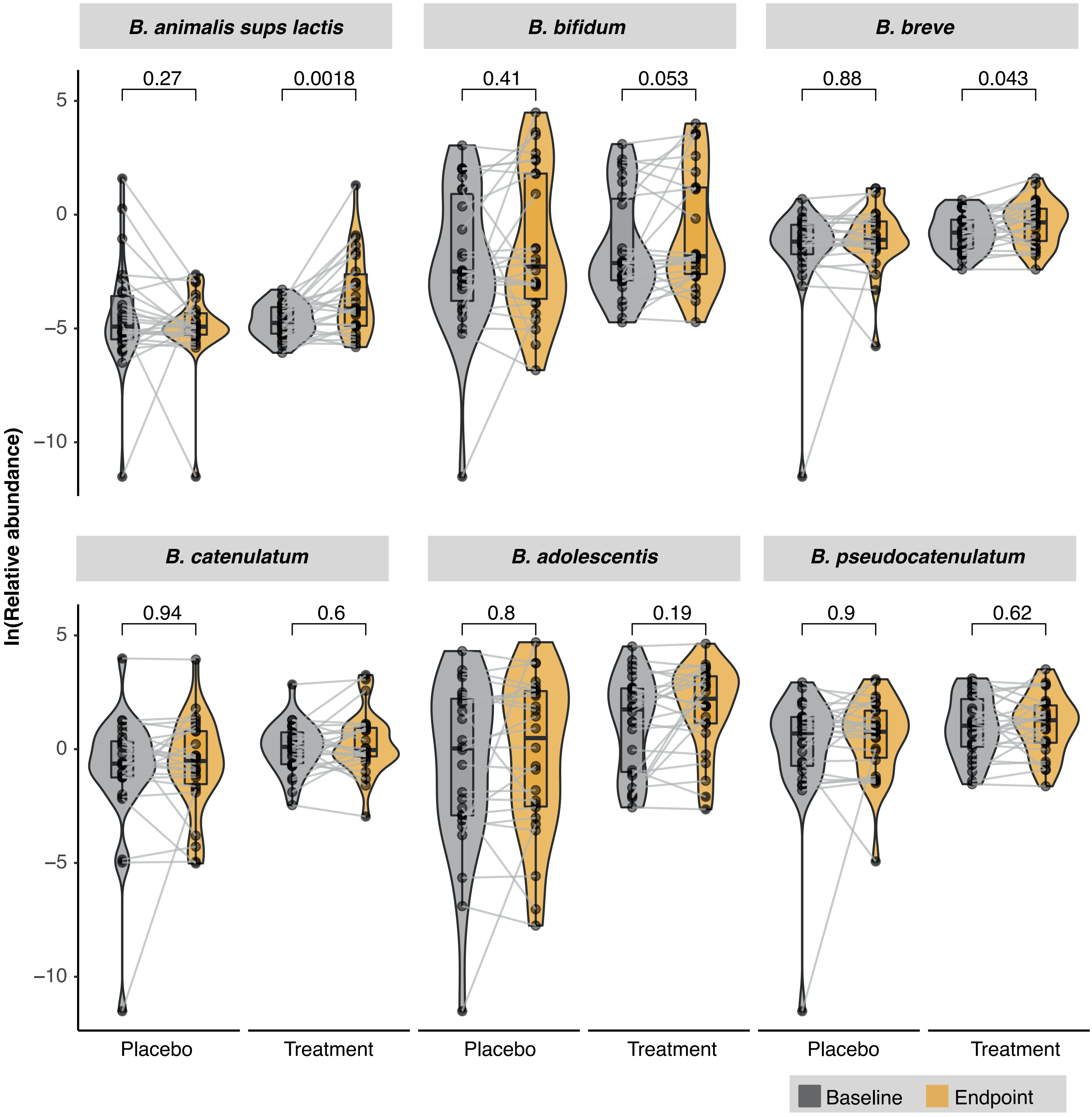

Supplemental Figure S2: Increase in bifidobacteria across timepoints and study groups. Detection of members of the genus Bifidobacterium at baseline and endpoint in the treatment vs placebo groups.
