# Supplementary material for: Functional response to a microbial synbiotic in the gastrointestinal system of children: a randomized clinical trial
Source: Pediatr Res. 2022 Nov 2;93(7):2005–13. doi: 10.1038/s41390-022-02289-0 (PMC10313516; doi:10.1038/s41390-022-02289-0)
Supplement: Supplementary file 4 — Supplementary Figure S3 [file 41390_2022_2289_MOESM4_ESM.pdf]

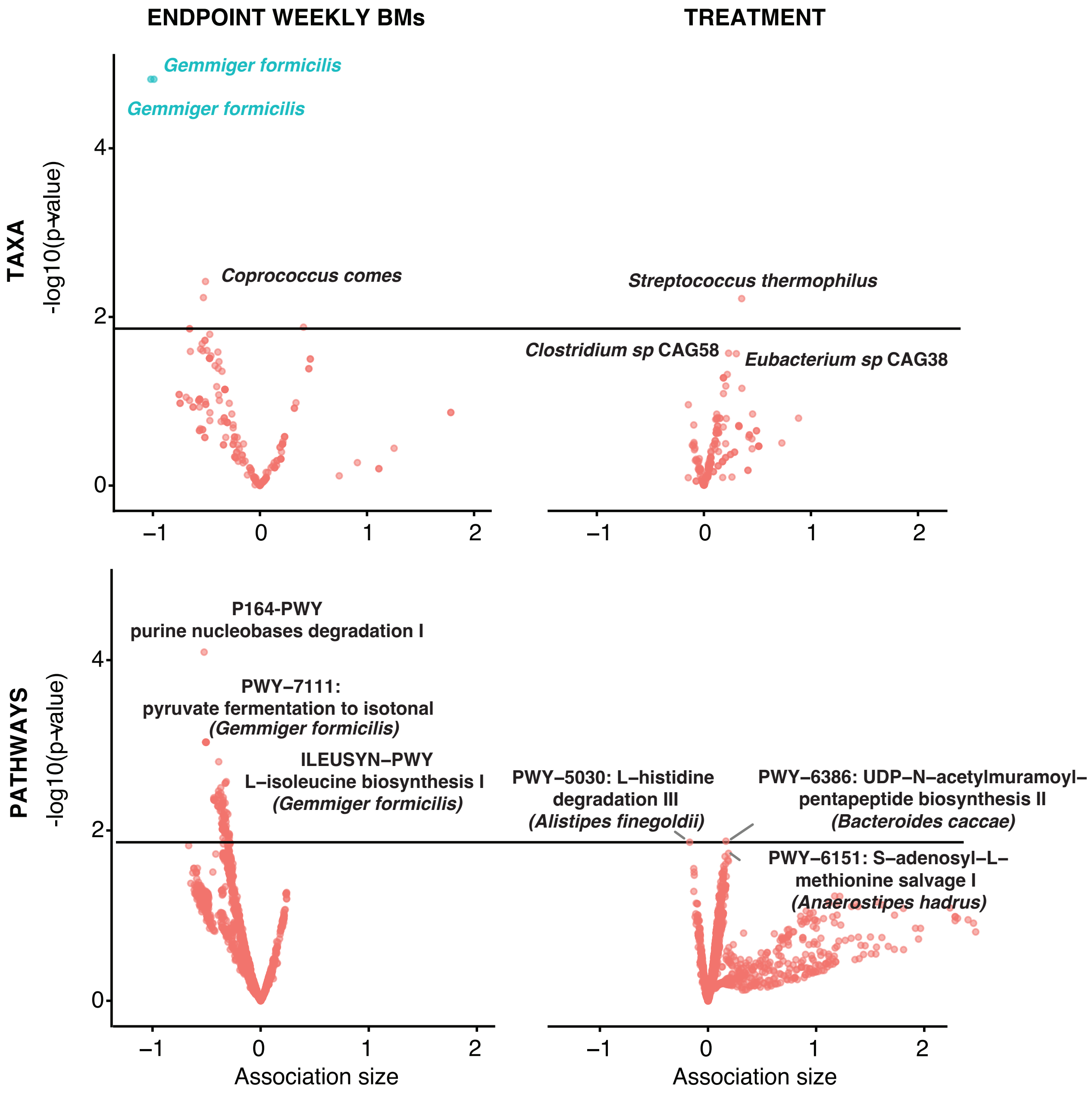

Supplemental Figure S3: Associations with treatment and endpoint WBMs. X-axes are beta-coefficients from linear regressions. Y axes are negative log10(q-values). Color denotes FDR-significance, the solid line indicates a nominal p-value = 0.05. The 3 most significant associations are reported for each plot.
