# Supplementary material for: Functional response to a microbial synbiotic in the gastrointestinal system of children: a randomized clinical trial
Source: Pediatr Res. 2022 Nov 2;93(7):2005–13. doi: 10.1038/s41390-022-02289-0 (PMC10313516; doi:10.1038/s41390-022-02289-0)
Supplement: Supplementary file 5 — Supplementary Figure S4 [file 41390_2022_2289_MOESM5_ESM.pdf]

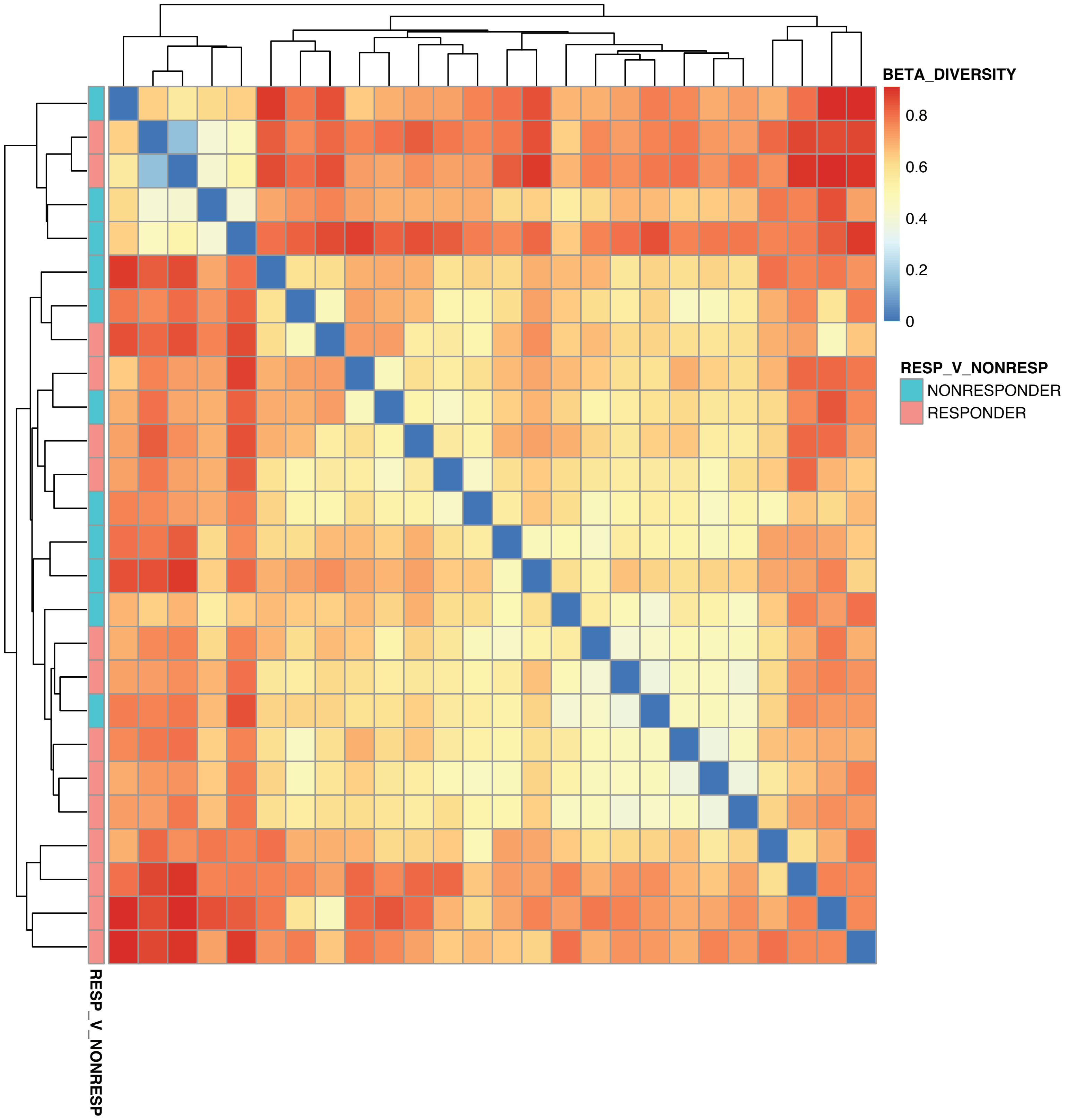

Supplemental Figure S4: Beta diversity between responders and non-responders. Each row/column represents a different patient.
