# Supplementary material for: Functional response to a microbial synbiotic in the gastrointestinal system of children: a randomized clinical trial
Source: Pediatr Res. 2022 Nov 2;93(7):2005–13. doi: 10.1038/s41390-022-02289-0 (PMC10313516; doi:10.1038/s41390-022-02289-0)
Supplement: Supplementary file 6 — Supplementary Figure S5 [file 41390_2022_2289_MOESM6_ESM.pdf]

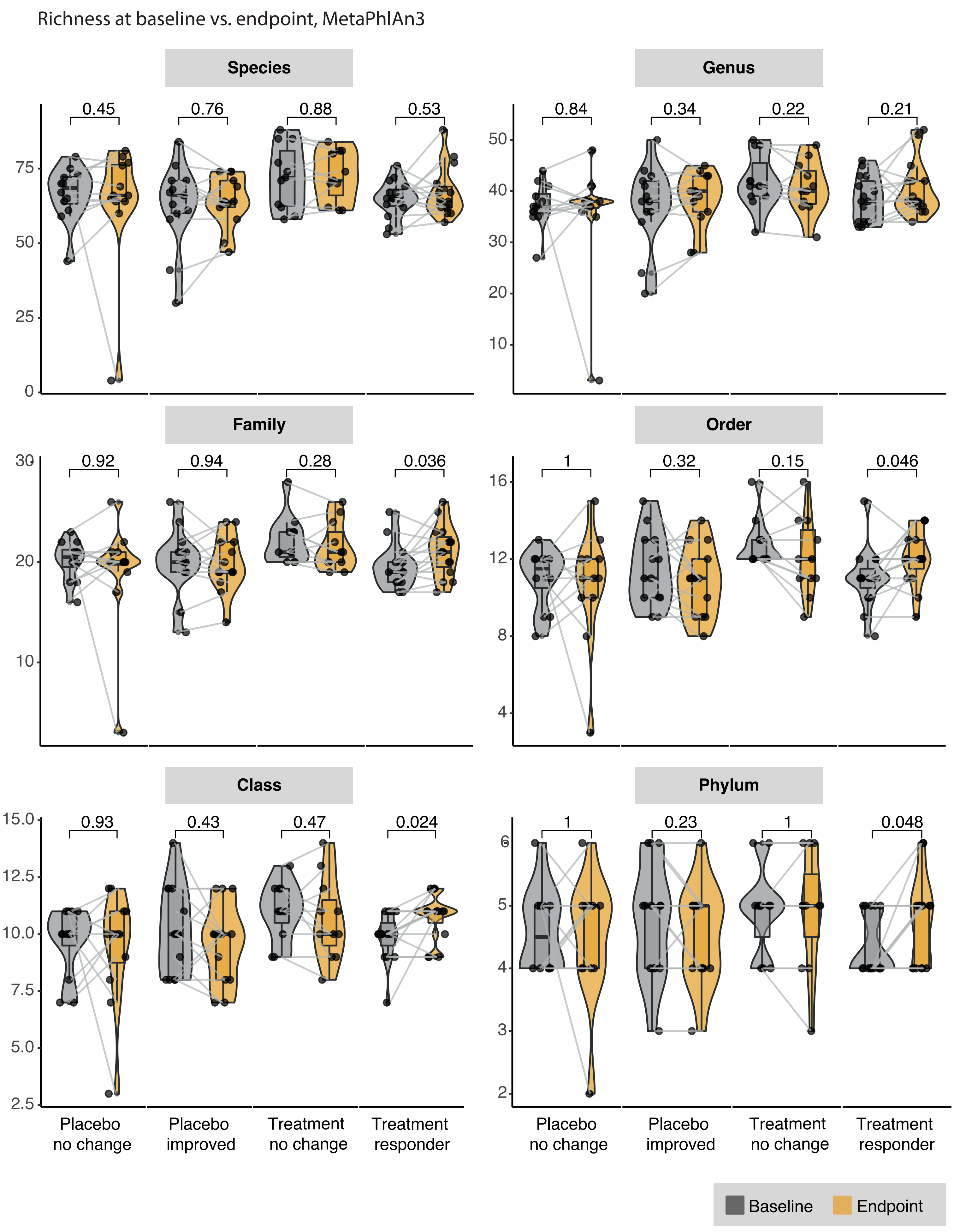

Supplemental Figure S5: Change in richness between baseline and endpoints for all individuals across all taxonomic levels.
